# Engineering the Outcoupling Pathways in Plasmonic Tunnel Junctions via Photonic Mode Dispersion for Low-Loss Waveguiding

**DOI:** 10.1021/acsnano.3c10832

**Published:** 2023-12-26

**Authors:** Zhe Wang, Vijith Kalathingal, Goki Eda, Christian A. Nijhuis

**Affiliations:** † Department of Electrical and Computer Engineering, National University of Singapore, 4 Engineering Drive 3, 117583, Singapore; ‡ Department of Chemistry, National University of Singapore, 3 Science Drive 3, Singapore 117543, Singapore; § Department of Physics, Kannur University, Swami Anandatheertha Campus-Payyanur, Kannur-670327, Kerala India; ∥ Department of Physics, National University of Singapore, 2 Science Drive 3, Singapore 117542, Singapore; ⊥ Centre for Advanced 2D Materials and Graphene Research Centre, National University of Singapore, 6 Science Drive 2, Singapore 117546, Singapore; # Hybrid Materials for Optoelectronics Group, Department of Molecules and Materials, MESA+ Institute for Nanotechnology and Center for Brain-Inspired Nano Systems, Faculty of Science and Technology, University of Twente, 7500 AE Enschede, The Netherlands

**Keywords:** Optical and plasmonic outcoupling, inelastic electron tunneling, tunnel junctions, multilayer substrate, silicon nitride photonics

## Abstract

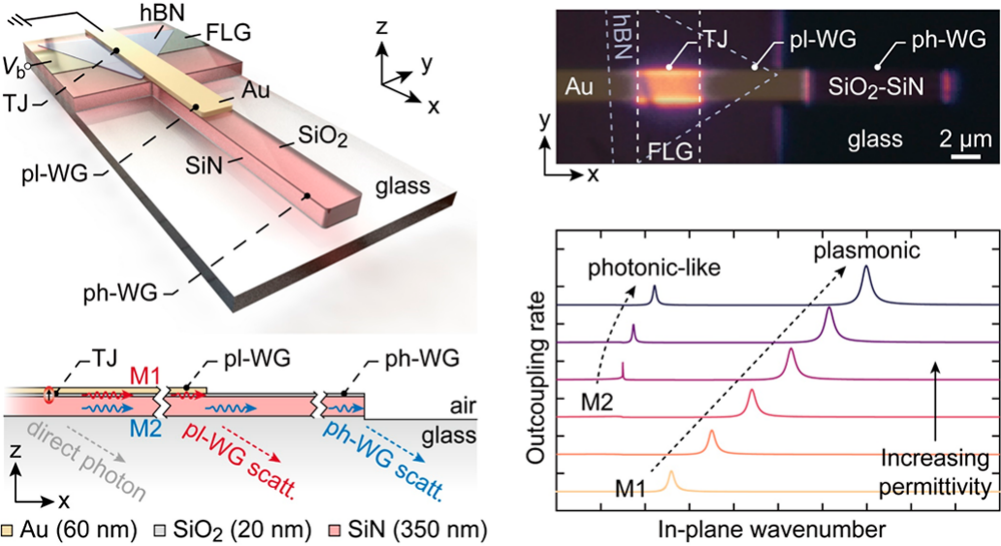

Outcoupling of plasmonic modes excited by inelastic electron tunneling (IET) across plasmonic tunnel junctions (TJs) has attracted significant attention due to low operating voltages and fast excitation rates. Achieving selectivity among various outcoupling channels, however, remains a challenging task. Employing nanoscale antennas to enhance the local density of optical states (LDOS) associated with specific outcoupling channels partially addressed the problem, along with the integration of conducting 2D materials into TJs, improving the outcoupling to guided modes with particular momentum. The disadvantage of such methods is that they often involve complex fabrication steps and lack fine-tuning options. Here, we propose an alternative approach by modifying the dielectric medium surrounding TJs. By employing a simple multilayer substrate with a specific permittivity combination for the TJs under study, we show that it is possible to optimize mode selectivity in outcoupling to a plasmonic or a photonic-like mode characterized by distinct cutoff behaviors and propagation length. Theoretical and experimental results obtained with a SiO_2_–SiN–glass multilayer substrate demonstrate high relative coupling efficiencies of (62.77 ± 1.74)% and (29.07 ± 0.72)% for plasmonic and photonic-like modes, respectively. The figure-of-merit, which quantifies the tradeoff between mode outcoupling and propagation lengths (tens of μm) for both modes, can reach values as high as 180 and 140. The demonstrated approach allows LDOS engineering and customized TJ device performance, which are seamlessly integrated with standard thin film fabrication protocols. Our experimental device is well-suited for integration with silicon nitride photonics platforms.

## 1 Introduction

It is well-known that inelastic electron tunneling (IET),^1^ where a quantum of energy  is lost during the transit, is associated with various radiative and nonradiative decay or outcoupling pathways. The quantum efficiency of IET energy transfer to radiation in the visible (Vis) or near-infrared (NIR) range is significant only if at least one of the electrodes for a tunnel junction (TJ) is plasmonically active. In principle, such plasmonic TJs allow for ultrafast excitation (on the quantum mechanical tunneling times scales on the order of femtoseconds) and low operating voltages (< 3 V) and are, therefore, interesting to explore as an alternative for applications that require electrical sources of photons and plasmons.^2−10^ However, TJs so far have a large number of outcoupling pathways leading to photon generation,^8,9^ surface plasmon polariton (SPP) excitation,^10−12^ and nonradiative modes such as phonons^13^ and vibronic excitations.^14,15^ But for applications, it is crucial to control the outcoupling pathways tailored for the respective target requirements.

When a bias voltage (*V*_b_) is applied across a TJ, tunneling processes are spatially localized to a subatomic region.^16^ Hence, the resulting IET can be seen as a dipole source^17^ that decays its power into all available outcoupling channels (modes),^8−15^ because of the large in-plane momentum (*k*_∥_) it can supply. The outcoupling strength to these individual modes is quantified by the local density of optical states (LDOS).^18−20^ Hence, it is important to carefully design the electromagnetic environment around the dipole to attain precise control over the outcoupling pathways. Previous attempts to achieve this control involve plasmonic antennas, typically with dimensions smaller than 100 nm, that have been integrated as an active^6−9^ or passive^21,22^ TJ component. This approach allows for selective enhancement of the LDOS associated with specific energy through localized plasmon resonance, thereby improving light emission efficiencies. Nanoscale surface roughness^23^ can also effectively generate outcoupling pathways but complicate such approaches. Furthermore, directional antennas^4,24,25^ can enhance the emission directivity, transforming the omnidirectional dipole emission into a unidirectional one.

For large-area metal–insulator–metal (MIM) configurations, where only broadband junction modes (MIM-SPPs) are directly excited by the IET, controlling the outcoupling pathways can be readily achieved by simply changing the geometry of the TJ^26,27^ or the thickness of the top or bottom electrode.^28^ This approach exploits outcoupling via mode scattering at TJ edges to the integrated slab waveguides,^29^ which supports single-interface SPP modes where, for example, the coupling efficiency to the extended waveguide can be improved by reducing the thickness of the metal electrodes,^28^ but at the cost of reduced LDOS and IET efficiencies. An intuitive way to improve the outcoupling from highly confined MIM-SPP modes is to replace one metal with graphene (G), which results in direct excitation of single-interface SPP modes,^22,30^ whose momentum is 10 times lower than that of the MIM-SPP mode, which is more suitable for mode dispersion engineering. In our prior report on metal–insulator-graphene (MIG) TJ devices,^30^ we achieved a relative coupling efficiency (*η*) of SPPs, accounting for up to 80% of the overall optical outcoupling (*Γ*_opt_, including SPPs and photons). Regrettably, the unilateral open configuration of MIG-TJs undermines the high LDOS associated with the MIM-SPP mode, leading to a reduced overall enhancement in optical outcoupling (*Γ*_opt_/*Γ*_0_, where *Γ*_0_ denotes the free space decay rate), relative to MIM-TJs.

For all of these reasons, alternative viable methods to engineer mode dispersion and tailor the outcoupling pathways without compromising the MIM or MIG multilayer characteristic of the TJs are required. It is also important to note that achieving a simultaneous combination of high outcoupling to guided modes and low mode propagation losses is crucial for optimizing TJs in long-distance waveguiding systems. High propagation losses restrict all-metal plasmonic circuitry and demand alternative options without an extensive compromise on the optical enhancement offered by the plasmonic components. In principle, a combination of high and low permittivity dielectric layers can be used to confine and guide low-loss electromagnetic modes in a planar geometry.^31−35^ Here, we integrate plasmonic MIG-TJs on a multilayered substrate that incorporates a specially designed combination of high and low permittivity dielectric layers connected to a photonic waveguide. In this way, we modified the plasmonic mode properties of the TJ to excite a photonic-like mode with a much larger propagation length (*l*_p_) than that of the single-interface SPPs. The high permittivity layer, positioned to support guided modes within the optical near field of the TJ, facilitates efficient energy transfer from the IET dipole into guided modes. To experimentally verify this point, we choose silicon nitride (SiN) and silicon dioxide (SiO_2_) as the constituent materials, since they provide a high contrast of dielectric permittivity and low absorption at optical frequencies.^36^ With a dedicated waveguide extended from the TJ area, the *η* values to the plasmonic ((62.77 ± 1.74)%) and photonic-like modes ((29.07 ± 0.72)%) are evaluated. The decent tradeoff between outcoupling strength (*ηΓ*_opt_/*Γ*_0_) to each individual optical mode and *l*_p_ results in reasonably high figure-of-merit^37,38^ (FOM = (*l_p_*/*λ*_0_*)ηΓ*_opt_/*Γ*_0_, where *λ*_0_ stands for the wavelength) of 180 and 140, respectively, which is a factor of 7–8 higher than those values of MIG-TJ on glass. Our demonstration provides a versatile method for engineering the LDOS to customize the TJ device’s performance that is readily compatible with the silicon nitride-based photonics platforms.

## 2 Results and Discussion

**Figure 1:**
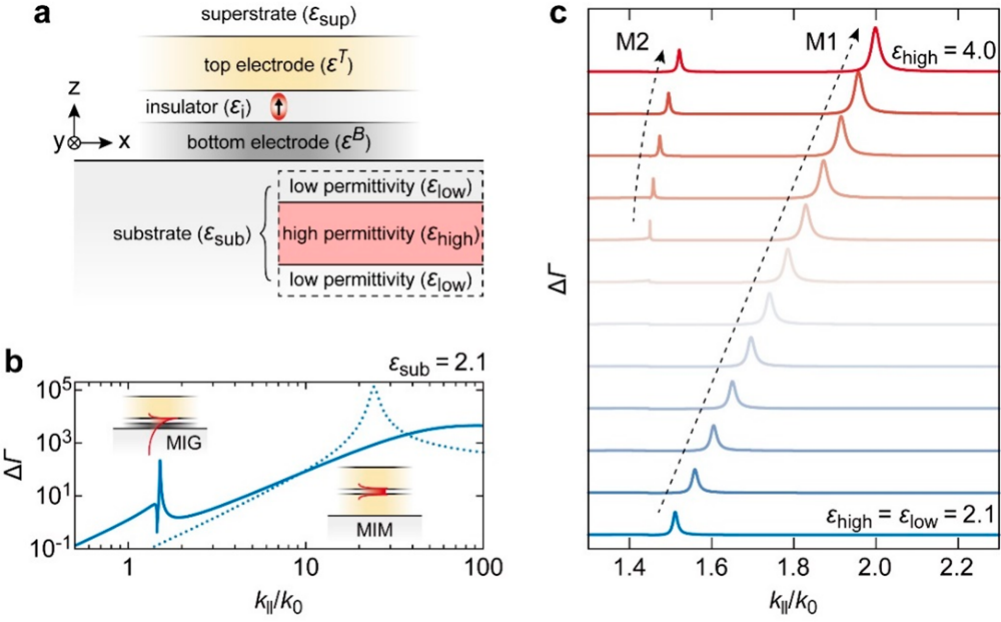
(a) Cross-sectional view of the TJs with two electrodes separated by a thin insulator used for modeling. The red oval represents the IET dipole, and the substrate and superstrate are homogeneous single layers extending to infinity. The inset shows the multilayer stack used in place of the homogeneous substrate to tailor the dipole outcoupling pathways. (b) Δ*Γ* plotted as a function of normalized in-plane wavenumber (*k*_||_/*k*_0_) for the MIG (solid blue line) and the MIM (dotted blue line) configurations on substrates with *ε*_sub_ = 2.1 (i.e., the permittivity of glass). The insets show schematic representations of the plasmonic modes supported by the two configurations. (c) Δ*Γ* calculated for MIG on a multilayer substrate with *ε*_high_ varied from 2.1 to 4.0 with a fixed *ε*_low_ value of 2.1. The thickness of the upper low permittivity layer is set at 20 nm, and that of the high permittivity layer is set at 350 nm in the calculations.

Figure 1a shows a schematic illustration of the TJ used in our calculations. Each TJ comprises two electrodes (*ε^T^* and *ε^B^*) separated by an insulator layer (*ε*_i_) with a thickness of 2 nm. The TJ is supported on a dielectric substrate (*ε*_sub_) or a multilayered substrate (inset), and the superstrate (*ε*_sup_) is normally air (*ε*_sup_ = 1). The red oval represents the IET dipole. From the dipole decay rate *Γ* (normalized to free space decay rate *Γ*_0_), the LDOS for various outcoupling channels can be quantified following the method previously reported.^18^ The differential of *Γ* with respect to *k*_∥_/*k*_0_ gives a more intuitive understanding of the mode-LDOS and is represented as ), where *k*_0_ is the free space wavenumber (see section S1 in the Supporting Information (SI) for details).^30^

To benchmark our approach, we first calculated the properties of the MIM and MIG junctions on a homogeneous substrate. Figure 1b shows a log–log plot of Δ*Γ* calculated for the MIM (dotted blue line) and MIG (solid blue line) configuration with infinitely thick Au layer as the top and infinitely thick Au or few-layer graphene (FLG) as the bottom electrode. The two sets of Δ*Γ* spectra exhibit peaks at *k*_||_/*k*_0_ ≈ 1.5 and 24, corresponding to the respective characteristic plasmonic modes supported by the MIG and the MIM configurations. We note that, in ΔΓ spectra, the inverse of the peak width determines the *l*_p_ of a mode.^39^ The MIM configuration yields a much broader peak, resulting in a much smaller *l*_p_ (tens of nm) than the MIG configuration, which offers a longer *l*_p_ of a few micrometers. While the MIG configuration features lower losses, the overall emission rate is 2–3 orders of magnitude lower than that of the MIM configuration, judged from the peak values of each configuration’s characteristic modes. These results are similar to earlier reports, but importantly, they demonstrate the tradeoff between lowering the *k*_||_/*k*_0_ and emission rates.

Now, we turn to the layered substrate to demonstrate control over the dispersion mode. A three-layer stack is considered, as illustrated in Figure 1a (inset), where the substrate is modified to *ε*_sub_ = *ε*_low_ – *ε*_high_ – *ε*_low_, where *ε*_low_ and *ε*_high_ represent low and high permittivity layers, respectively. For representation purposes, we set the thickness of the upper low permittivity layer at 20 nm and that of the high permittivity layer at 350 nm. In general, the thicknesses of these layers are important parameters in controlling the mode dispersion and propagation length. This is investigated further in detail to understand the characteristics of the modes supported by the layered substrate. The immediate effect of this modification on dipole outcoupling is evident in the Δ*Γ* plot (Figure 1c), where we fix the *ε*_low_ at 2.1 to match *ε*_glass_ while varying the *ε*_high_ from 2.1 to 4. When *ε*_low_ = *ε*_high_ = 2.1, the mode dispersion is identical to that of the MIG case shown in Figure 1b (solid blue line), where a single mode (M1) is observed. As *ε*_high_ is progressively increased to 4, *k*_∥_/*k*_0_ of M1 monotonically shifts from 1.51 to 2.00, indicating higher mode confinement. Interestingly, a second mode (marked as M2) at a lower *k*_∥_/*k*_0_ (∼1.52) also emerges with the increase in *ε*_high_, with a narrower peak width than M1, indicating a lower propagation loss. Apparently, M2 is less sensitive to *ε*_high_ than M1.

**Figure 2:**
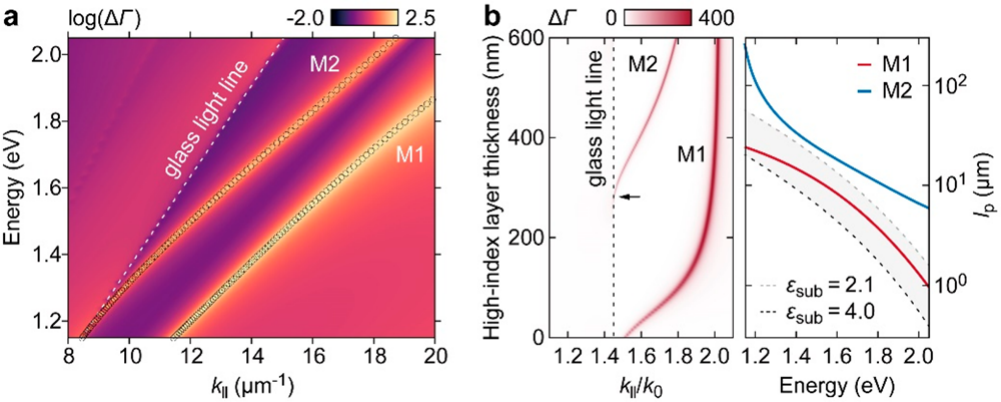
(a) log(Δ*Γ*) plotted as a function of *k*_||_ and energy. M1 and M2 modes are demarcated with hollow black circles at the intensity maxima. The dashed white line indicates the light dispersion in the glass medium. (b) The left panel shows Δ*Γ* evaluated as a function of *k*_||_/*k*_0_ and *ε*_high_ layer thickness at an energy of 1.37 eV (≡ 900 nm). The black arrow indicates where the M2 dispersion crosses the light line in the glass (dashed vertical line). The right panel shows propagation lengths *l*_p_ evaluated for M1 (solid red line) and M2 (solid blue line) as a function of energy. The *l*_p_ variation for Au single-interface SPP modes with *ε*_sub_ = 2.1 (dashed gray line) to *ε*_sub_ = 4.0 (dashed black line) is shown as a shaded area.

To improve our understanding of the characteristics of M1 and M2, Δ*Γ*, which is calculated as a function of *k*_∥_ and energy, is plotted in Figure 2a. The Δ*Γ* is plotted on a log scale and exhibits two bright bands corresponding to the dispersion of M1 and M2 modes, with their intensity maxima indicated by hollow black circles. Both modes are located to the right of the light dispersion in glass (dashed white line), revealing their bound character. Since M1 and M2 show different sensitivities to the value of *ε*_high_, the true character of these modes is understood from the variation of effective indices with the thickness of the *ε*_high_ layer, as shown in Figure 2b (left panel). A distinct feature exhibited by M2 is the mode cutoff, where the dispersion curve intersects the glass light line (dashed vertical line) when the thickness of the high permittivity layer is 280 nm (indicated by the black arrow). This shows the photonic character of M2 compared to M1, which has no lower cutoff limit and, therefore, is plasmonic.

In the right panel of Figure 2b, we compare the *l*_p_ of M1 (solid red line) and M2 (solid blue line) for a *ε*_high_ layer thickness of 350 nm (larger than the M2 cutoff thickness) for the energy range of 1.2–2.0 eV. For the low permittivity layer between the MIG-TJ and the *ε*_high_ layer, a thickness of 20 nm is chosen, which can readily be formed during device fabrication (thinner films risk being inhomogeneous). Compared to the plasmonic mode M1, M2 shows more than a half order of magnitude improvement in *l*_p_, consistent with the photonic character of M2. As opposed to pure photonic waveguide cases, *l*_p_ of M2 is limited to ∼ 36 μm due to the interaction losses of M2 with the Au layer at the nanoscale proximity (∼ 20 nm). The shaded region in Figure 2b indicates the *l*_p_ of Au interface SPPs when the adjacent dielectric permittivity is varied in the range of 2.1 ≤ *ε*_sub_ ≤ 4.0. We note that M1 falls in this range and exhibits an intermediate *l*_p_ value, compared to single-interface SPP modes. The photonic-like M2, while experiencing loss, falls out of the shaded zone, demonstrating an improved *l*_p_ value, compared to plasmonic modes.

**Figure 3:**
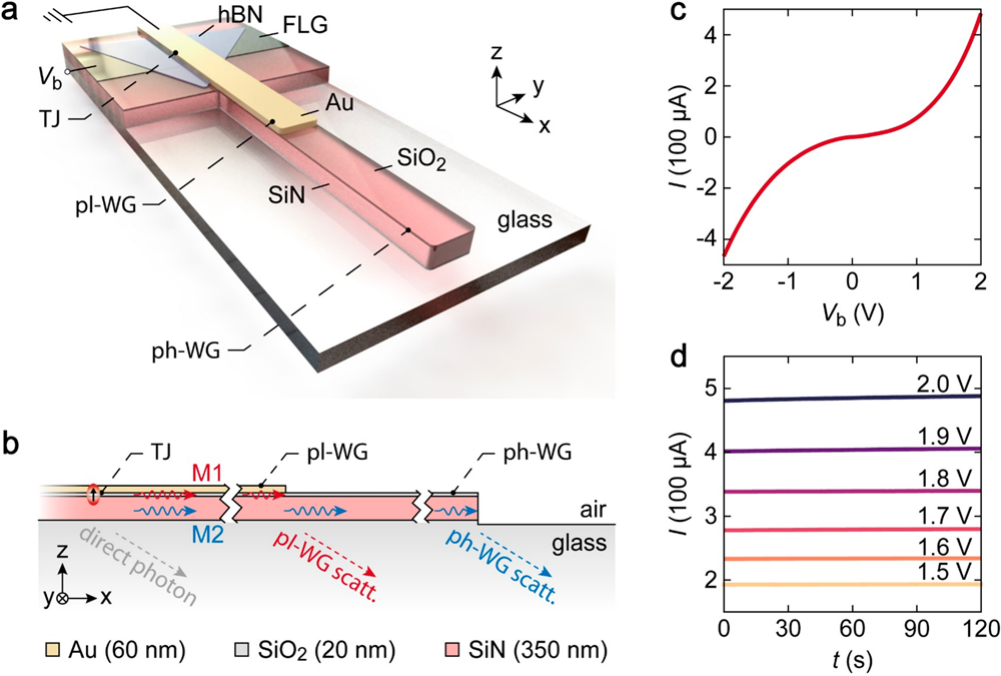
(a) Schematic illustration of the MIG-TJ on a SiO_2_–SiN–glass multilayer substrate. The Au and SiO_2_–SiN strips extend from the TJ area, serving as pl-WG and ph-WG. (b) Cross-sectional (*xz*-plane) view of the device. The red oval at the TJ area’s center represents the effective IET dipole. (c) Experimental *I*(*V*_b_) curve measured for the range of |*V*_b_| ≤ 2 V. (d) *I*(*t*) traces recorded over 120 s for *V*_b_ = 1.5–2.0 V.

Figure 3a shows the schematic of the experimental device with MIG-TJ on a SiO_2_–SiN–glass multilayer substrate with *ε*_SiN_ = 4.0 and *ε*_SiO_2__ = 2.1, providing a high permittivity contrast with low absorption.^36^ SiN and SiO_2_ are chosen for the multilayer substrate as these materials are readily available along with well-established fabrication processes.^40^ The fabrication details are described in the Methods section. In the design, an Au strip is used as one of the electrodes of TJ, which also serves as the plasmonic waveguide (pl-WG) that extends up to 10 μm from the TJ. The SiO_2_–SiN stack is etched to create a strip extending beyond the Au strip’s length. The SiO_2_–SiN-glass combination, characterized by its high permittivity contrast, fulfills the requirements for total internal reflection, forming a photonic waveguide (ph-WG; see Figure 3b). The two-strip design extending from the TJ area allows us to independently analyze the propagation and scattering of the M1 and M2 modes from far-field optical measurements.

**Figure 4:**
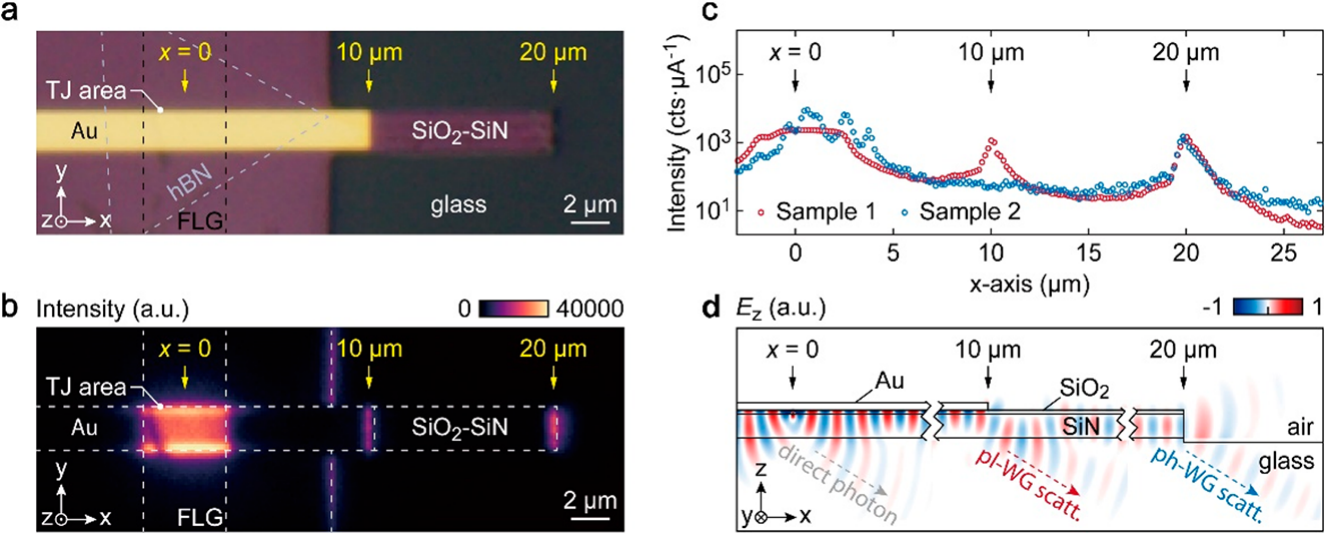
(a) Optical microscope image of the experimental device. Dashed lines outline the FLG and hBN. (b) EMCCD image of light emission for *V*_b_ = 1.5 V. The emission intensity was integrated for 30 s during light collection. (c) Emission intensity integrated along the width (y) of the waveguides plotted as a function of *x* for Sample 1 (hollow red circles) and Sample 2 (hollow blue circles). Au strip is etched out in the case of Sample 2. Both datasets are normalized to the average tunnel current. (d) Simulated electric field (*E*_*z*_) profile in the *xz*-plane from FDTD method. The dipole source is positioned at *x* = 0 on the *x*-axis and 1 nm below the Au layer in the *z*-direction.

The electrical bias triggers tunneling processes in the MIG-TJ area. As described in our previous report,^30^ the TJ area containing IET dipole excites all guided modes and emits photons directly (dashed gray arrow, Figure 3b). The propagations of M1 and M2 originating from the IET dipole are represented by wavy red and blue arrows. At the pl-WG end, M1, which is essentially plasmonic, experiences severe impedance mismatch due to the termination of the Au strip, and most M1 power is scattered into the glass medium immediately (dashed red arrow, Figure 3b). In comparison, the photonic-like M2 is ideally suited to propagate in the ph-WG due to its photonic nature and matching impedance characteristics. It readily propagates until the end of the waveguide, resulting in M2 scattering (dashed blue arrow, Figure 3b). Figure 3c provides the current–voltage *I*(*V*_b_) curve of the experimental MIG-TJ to excite the guided modes (see Figure 4 for device images). The MIG-TJ used in our experiments exhibits good electrical stability, as is evident from the current–time (*I*(*t*)) traces displayed in Figure 3d (along with additional *I*(*V*_b_) scans in Figure S1 in the SI). This can be attributed to the electrical stability of the hBN barrier.^41^

Figure 4a shows the optical microscopy image of the fabricated device. The MIG-TJ comprises layers of Au (60 nm thick), hexagonal boron nitride (hBN, outlined by dashed gray lines), and FLG (outlined by dashed black lines). The TJ area is the overlapping junction between the Au and FLG electrodes. The *x*-coordinate for the TJ area center is defined as *x* = 0 (as indicated by the yellow arrow). Both the Au and SiO_2_–SiN waveguides have a width of 2 μm. We employed an inverted optical microscope with an electron multiplying charge-coupled device (EMCCD) camera to record the far-field emission from the device—all emission contributions, viz. M1 and M2 scattering (from *x* = 10 and 20 μm) and direct TJ area emissions are collected through the glass substrate using an index-matching oil immersion objective (NA = 1.49). Figure 4b shows the EMCCD image of the light emission for *V*_b_ = 1.5 V. For IET, in general, the maximum energy of the emitted photons is dictated by the quantum cutoff  and for the EMCCD, the lower detection limit is ∼1100 nm (∼1.1 eV). Therefore, in Figure 4b, with *V*_b_ = 1.5 eV (≡ 825 nm), the EMCCD detects photons in the range of 825–1100 nm (see the Methods section for measurement details). We observe that the direct emission from IET uniformly illuminates the TJ area. At the same time, the plasmonic mode (M1), which is generally nonradiative and confined to the metal–dielectric interface, scatters from the ±*y* edges of the Au strip and results in an enhanced intensity at these locations.^22,29,30^

Figure 4b shows the light scattering from the pl-WG and the ph-WG ends at *x* = 10 and 20 μm, respectively. Interestingly, we also note that the etched boundaries of the SiO_2_–SiN substrate along the *y*-axis show a weak scattering contribution, probably from M2, which isotropically propagates in all directions from the TJ before it couples to the ph-WG. To confirm the separate excitation of M1 and M2 from the TJ, we compare the emission characteristics of the first device shown in Figure 4b (Sample 1) with a second MIG-TJ (Sample 2) lacking the pl-WG but with the photonic SiO_2_–SiN strip (see section S3 in the SI). Emission intensities, integrated along the width (y) of the waveguides and normalized to the average tunnel current, are plotted as a function of *x* in Figure 4c for Sample 1 (red circles) and Sample 2 (hollow blue circles). At *x* = 0 and *x* = 20 μm, emission from both Sample 1 and Sample 2 shows similar characteristics. However, Sample 2 shows no signature of the plasmonic scattering (M1) peak at *x* = 10 μm. It is worthwhile noting that M2 propagation remains unaffected, extending up to *x* = 20 μm, and consequently, the M2 scattering at *x* = 20 μm exhibits nearly equal intensities for both samples, corroborating the independent excitation of M1 and M2 from the TJ area.

We used two-dimensional finite-difference time-domain (FDTD) simulations to verify the experimental observations qualitatively. A vertical dipole with broadband spectral response located at *x* = 0 (Figure 4d) is used as the source for exciting M1 and M2 modes. Figure 4d demonstrates the simulated electric field (*E*_*z*_) distribution, which reveals direct emission to the glass substrate and excitation of propagating modes within the dedicated waveguides. As the Au strip terminates at *x* = 10 μm, the plasmonic M1 undergoes scattering, whereas the photonic-like M2 propagates along the SiO_2_–SiN waveguide until it reaches *x* = 20 μm. The IET dipole at *x* = 0 eventually results in all three distinct emissions observed at *x* = 0, 10, and 20 μm. The simulation results are in good agreement with the experimental observations.

**Figure 5:**
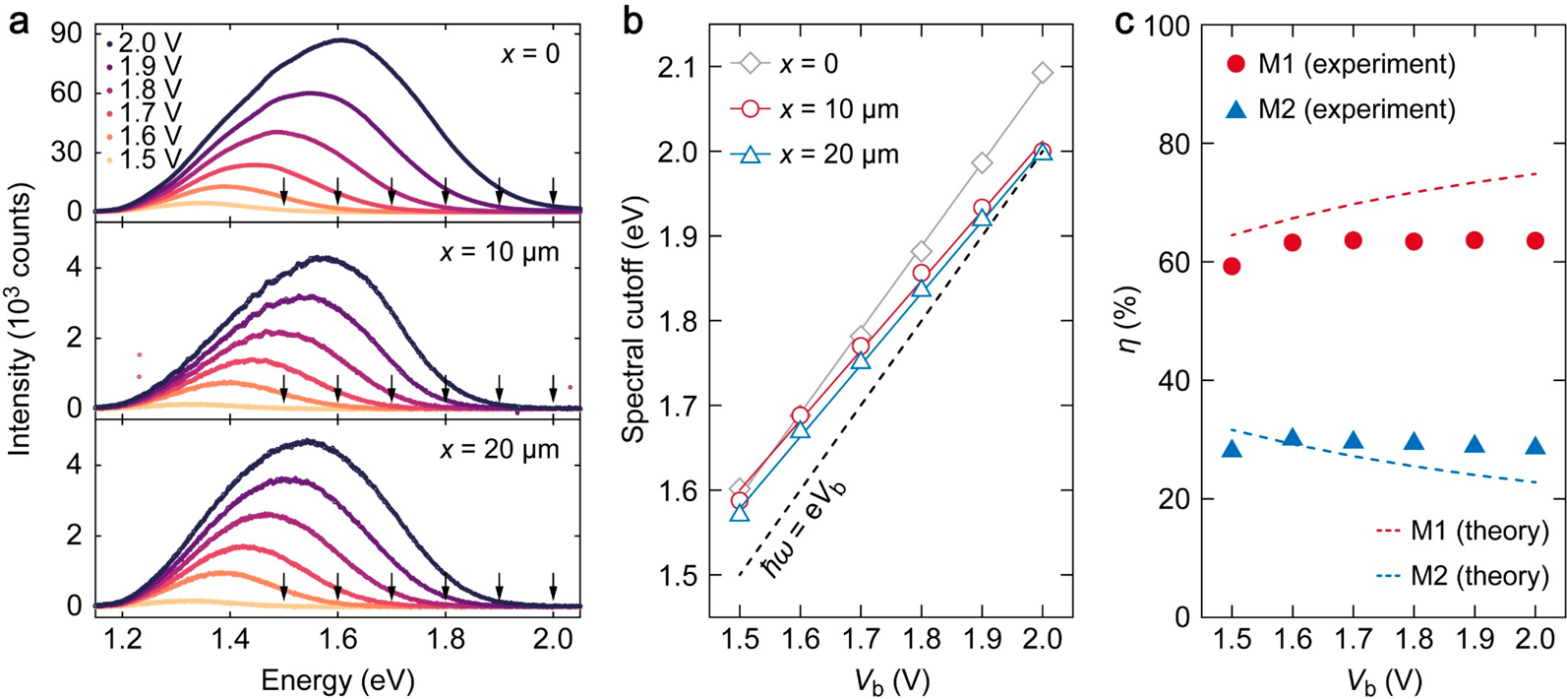
(a) Light emission spectra collected from TJ area at *x* = 0 (top panel), from Au strip end at *x* = 10 μm (middle panel), and from SiO_2_–SiN strip end at *x* = 20 μm (bottom panel). The spectra are integrated over 120 s. The black arrows indicate the quantum cutoff for energy. (b) Cutoff energies as a function of *V*_b_ for the light emission spectra collected for *x* = 0 (hollow gray diamonds), *x* = 10 μm (hollow red circles), and *x* = 20 μm (hollow blue triangles). Solid lines represent the linear fit to the experimental data. The dashed solid line represents the theoretical cutoff .) (c) Relative coupling efficiency *η* for M1 (solid red circles) and M2 (solid blue triangles), as a function of *V*_b_, calculated from experiments. Theoretical data are depicted in the dashed red line for M1 and the dashed blue line for M2.

We analyzed the spectral characteristics of emission from *x* = 0 (direct emission), *x* = 10 μm (plasmonic), and *x* = 20 μm (photonic) with a spectrograph (for the range 300–1100 nm). Figure 5a shows the light emission spectra recorded for all three locations. All spectra exhibit a broadband emission with quantum cutoff , indicated by black arrows) as the high energy limit. As *V*_b_ increases from 1.5 to 2.0 V, the emission intensities increase, accompanied by a blueshift for the spectral cutoff. We employ Gaussian functions for spectral fitting to determine the cutoff energies. The cutoff energy is defined as the value at 3σ of the Gaussian function. Figure 5b illustrates the correlation between the spectral cutoff and *V*_b_. It is evident that, with increasing *V*_b_, the spectral cutoff energies for all three collection positions (*x* = 0 (denoted by hollow gray diamonds), *x* = 10 μm (denoted by hollow red circles), and *x* = 20 μm (denoted by hollow blue triangles)) experience a blue shift and remain closely aligned with the theoretical value (indicated by the dashed black line), although they are slightly higher likely due to the influence of finite lattice temperature.^42^ Additionally, note that the experimental spectral cutoff observed at *x* = 0 (corresponding to direct photon emission) consistently surpasses the theoretical cutoff by ∼80 meV. In contrast, as *V*_b_ increases, the spectral cutoff energies for positions *x* = 10 and 20 μm gradually converge toward the theoretical values, as evident by the linear fits to the experimental data (solid color lines). This convergence trend can be attributed to the gradual increase in propagation loss as the energy of guided modes approaches 2 eV. This experimental observation aligns with the theoretical analysis in Figure 2b (right panel).

We integrate the spectral intensities to determine the *η* of M1 and M2 by comparing them with the direct photon emission from the TJ area (refer to section S4 in the SI for details). Figure 5c shows the *η* values as a function of *V*_b_ in the range of 1.5–2.0 V. The average *η* in this range for M1 (solid red circles) reaches (62.77 ± 1.74)%, while M2 (solid blue triangles) reaches (29.07 ± 0.72)%. The experimental values agree well with the theoretical estimates (dashed red line for M1, dashed blue line for M2) based on an integrated Δ*Γ* calculation (refer to sections S1 and S4 in the SI for details). We note that, in ref (30), the *η* value of the plasmonic mode in bare MIG-TJs is ∼80%. This contrast underscores that the multilayer design enables us to preferentially outcouple the photonic-like mode (M2) at the expense of the plasmonic mode (M1).

Furthermore, we invoke the FOM^37,38^ to quantify the tradeoff between the mode outcoupling and the propagation losses. Table 1 provides the theoretical parameters for M1 and M2, including *ηΓ*_opt_/*Γ*_0_ and *l*_p_ obtained from Figures 2a and 2b, respectively. These values result in an FOM of M1 of 180 and that of M2 of 140. The FOM values are evaluated for the central wavelength of the experimental spectra (*λ*_0_ ≈ 900 nm when *V*_b_ = 1.5 V; see Figure 5a). Applying the same approach, we determined a FOM of 23 for the single-interface SPP mode in the bare MIG configuration, with *ηΓ*_opt_/*Γ*_0_ = 4 and *l*_p_ = 5.2 μm.^30^ This indicates a significant improvement in overall device performance when hybridizing MIG TJs with multilayer substrates, compared to that with the previously reported bare MIG configurations. Despite the improvement, it is essential to acknowledge that there remains room for refining the tradeoff between outcoupling and losses. Achieving this improvement may necessitate further optimizations of the thickness or permittivity of the substrate layers. It would be interesting to explore alternative dielectric films characterized by low loss and high permittivity, such as TiO_2_ or Al_2_O_3_.^43^ However, the chance of eliminating propagation losses by relying solely on modifying the multilayer substrate of the TJ is limited. To further narrow the divide between plasmonic TJs and photonic transmission lines, one can incorporate other structural designs, including techniques like phase matching^44,45^ or adiabatic conversion^46,47^ of the outcoupled modes.

**Table 1:** Theoretical Values of the FOM for M1 and M2

mode	*ηΓ*_opt_/*Γ*_0_	*l*_p_ (μm)	FOM
M1	10.48	15.52	180
M2	3.45	36.68	140

## 3 Conclusions

In conclusion, our study demonstrates a method to engineer the LDOS of MIG-TJs by modifying the SiN and SiO_2_ multilayer substrate. By precisely controlling the dielectric permittivity of the constituent layers, we effectively modify the outcoupling channels of the MIG-TJ and the properties of the propagating modes. The junctions were equipped with dedicated waveguides extending from the TJ area, enabling us to evaluate the *η* values of different guided modes. Our experimental results show that outcoupling from the TJ to the plasmonic and photonic-like modes can reach (62.77 ± 1.74)% and (29.07 ± 0.72)%. The decent tradeoff between outcoupling and propagation losses for the plasmonic and photonic-like modes results in reasonably high FOM values of 180 and 140, respectively, a factor of 7–8 improvement over MIG-TJs lacking the multilayered structure, showcasing that our approach offers a versatile method for tailoring the LDOS to customize the performance of plasmonic TJs. The experimentally demonstrated coupling of the photonic-like mode to lossless waveguides presents a promising opportunity to establish a connection between plasmonic TJs and photonic transmission lines.

## 4 Methods

### Fabrication

The multilayer substrates were prepared using PECVD (Oxford, Model PlasmaPro System100). A borosilicate glass substrate (Marienfield, 160 μm thick) was sequentially coated with a 350-nm-thick SiN layer and a 20-nm-thick SiO_2_ layer. MIG-TJs were fabricated on as-prepared SiO_2_–SiN-glass multilayer substrates. The FLG (∼1.6 nm, corresponding to five layers) and hBN (∼1.7 nm, corresponding to five layers) flakes were exfoliated from graphite (NGS Naturagraphit GmBH) and hBN (HQ Graphene) crystals. The flakes were assembled via the PMGI/PMMA sacrificial layer transfer method.^30^ The FLG flake was etched as a 5-μm-wide strip using electron beam lithography (EBL) (JEOL, Model JBX-6300FS) and O_2_ plasma etching (Femto Science, Model VITA). The hBN-FLG heterostructure was annealed in a vacuum at 220 °C for 6 h. The flake thicknesses were determined by using atomic force microscopy (AFM) (Bruker FastScan). A 2-μm-wide and 60 nm-thick Au strip, with a 2 nm-thick Ti adhesion layer, was then fabricated on the top of the hBN-FLG heterostructure using EBL and thermal evaporation (Kurt J. Lesker, Model NANO 36). The SiO_2_–SiN waveguide that extended from the Au strip was defined by etching the substrate 420 nm in depth using EBL and deep reactive ion etching (DRIE) (Oxford, Model PlasmaPro 100 Cobra).

### Measurements

The experimental characterizations were performed on an inverted optical microscope (Nikon, Model Eclipse Ti-E) with an oil-immersed objective (Nikon, 100× , NA 1.49). Continuous *V*_b_ supplied from a source meter (Keithley 6430) was applied to the sample through microprobes. The light emission of the device was collected from the backside of the sample through the glass substrate. Light emission images were collected using an electron-multiplying charge-coupled device (EMCCD) (Andor, Model iXon Ultra 897) with an integration time of 30 s. Light emission spectra were collected using a spectrometer (Andor, Model Shamrock 303i), with an integration time of 120 s. See Figure S3 in the SI for the quantum efficiency of the EMCCD.

### Numerical Simulation

The decay rate (*Γ*) is numerically evaluated in MATLAB, based on the theory given in section S1 in the SI. The differential of *Γ* with respect to *k*_||_/*k*_0_ is calculated for the stratified medium with an optical permittivity combination *ε*_sup_ – *ε^T^* – *ε*_i_ – *ε^B^* – *ε*_sub_ and the IET dipole is located in *ε*_i_. To obtain Figure 4d, we performed two-dimensional simulations using Lumerical FDTD Solutions, Ansys Canada, Ltd.^48^ The optical properties of Au and SiO_2_ were taken from Palik,^49^ and the SiN parameters were taken from Philipp.^50^ The total simulation area was 30 μm × 5 μm (*x* × *z*), with perfectly matched layers as all simulation boundaries. A minimum mesh size of d*x* = d*z* = 5 nm was applied across the simulation area.

## Supporting Information Available

Decay rate calculations; additional *I*(*V*_b_) scans; results for Sample 2 without Au strip extension; coupling efficiency calculations for M1 and M2; quantum efficiency plot of the EMCCD (PDF)
