# Supplementary material for: Engineering the Outcoupling Pathways in Plasmonic Tunnel Junctions via Photonic Mode Dispersion for Low-Loss Waveguiding
Source: ACS Nano. 2023 Dec 26;18(1):1149–56. doi: 10.1021/acsnano.3c10832 (PMC10786162; doi:10.1021/acsnano.3c10832)
Supplement: Supplementary file 1 — nn3c10832_si_001.pdf [file nn3c10832_si_001.pdf]

SUPPLEMENTARY INFORMATION

Engineering the outcoupling pathways in plasmonic tunnel junctions via photonic mode dispersion for low loss waveguiding

Zhe Wang,[†,1,2] *Vijith Kalathingal,*[†,1,3,*] *Goki Eda,*[2,4,5] *Christian A. Nijhuis*[6,*]

[1]Department of Electrical and Computer Engineering, National University of Singapore, 4 Engineering Drive 3, 117583, Singapore

[2]Department of Chemistry, National University of Singapore, 3 Science Drive 3, Singapore 117543, Singapore

[3]Department of Physics, Kannur University, Swami Anandatheertha Campus-Payyanur, Kannur-670327, Kerala, India

[4]Department of Physics, National University of Singapore, 2 Science Drive 3, Singapore 117542, Singapore

[5]Centre for Advanced 2D Materials and Graphene Research Centre, National University of Singapore, 6 Science Drive 2, Singapore 117546, Singapore

[6]Hybrid Materials for Opto-Electronics Group, Department of Molecules and Materials, MESA+ Institute for Nanotechnology and Center for Brain-Inspired Nano Systems, Faculty of Science and Technology, University of Twente, 7500 AE Enschede, The Netherlands

[†] These authors contributed equally.

* Author to whom correspondence should be addressed: email: vijith.k@kannuruniv.ac.in; c.a.nijhuis@utwente.nl

Section S1. Decay rate calculation

The decay rate (Γ) as a function of energy can be given by the dyadic Green's function $\overleftrightarrow{\boldsymbol{G}}(\boldsymbol{r}_m, \boldsymbol{r}_m, \omega)$ as[1, 2]

$$\frac{\Gamma}{\Gamma_0} = \frac{6\pi c}{\omega_0}\left[\boldsymbol{n}_\mu \cdot \mathrm{Im}\left\{\overleftrightarrow{\boldsymbol{G}}(\boldsymbol{r}_m, \boldsymbol{r}_m, \omega)\right\} \cdot \boldsymbol{n}_\mu\right] \tag{S1}$$

where $\boldsymbol{n}_\mu$ is the unit vector in the direction of the dipole moment ($\boldsymbol{\mu}$) for a dipole located at $\boldsymbol{r}_m$, $\Gamma_0 = \omega_0^3|\boldsymbol{\mu}|^2/(3\pi\varepsilon_0\hbar c^3)$ is the decay rate for free space. The normalized decay rate Γ/Γ_0 for the layered system $\varepsilon_{\text{sup}} - \varepsilon^T - \varepsilon_{\text{i}} - \varepsilon^B - \varepsilon_{\text{sub}}$ is calculated using angular spectrum representation of Γ in momentum space ($k_{||}/k_0$).[1] This yields the decay rate spectrum $\Delta\Gamma = 1/\Gamma_0 \cdot d\Gamma/d(k_{||}/k_0)$. Crucially, the calculation for Γ/Γ_0 involves the dyadic Green's function $\overleftrightarrow{\boldsymbol{G}}(\boldsymbol{r}_m, \boldsymbol{r}_m, \omega)$, which is evaluated from the reflection of individual plane waves at each of the interfaces of the stratified medium. For Figures 1b and 1c, $\Delta\Gamma$ is evaluated at a specific energy value (~ 1.4 eV), while in Figure 2a, $\Delta\Gamma$ is represented as a heatmap in $k_{||}$ and energy coordinates.

Section S2. Additional $\boldsymbol{I(V_\mathrm{b})}$ scans

Our device exhibits good electrical stability, as depicted in Figures 3c and 3d of the main text, maintaining stability throughout the experiment. In Figure S1, $I(V_\mathrm{b})$ scans recorded before (red squares) and after (blue triangles) the experiment are presented. All data, encompassing images and spectra, were collected over 4 days, during which no notable degradation due to aging was observed. The $I(V_\mathrm{b})$ data scans V_b range from 0 to +2 V, then to -2 V, and return to 0.

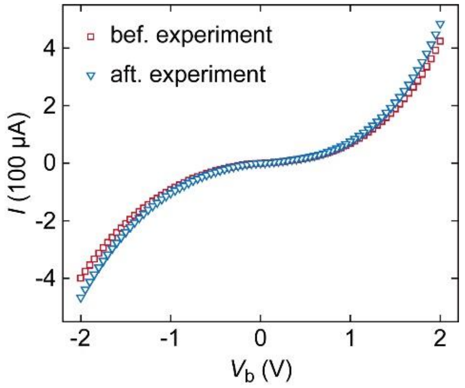

Figure S1. $I(V_b)$ scans recorded before and after the experiment. Red squares represent the $I(V_b)$ data collected after fabrication is complete (day 1), while blue triangles correspond to that collected after acquiring the full dataset, including images and spectra (day 4). $I(V_b)$ data sets were scanned from 0 to +2 V, then to -2 V, then back to 0."

Section S3. Results for Sample 2 without Au strip extension

To compare with Sample 1 (shown in main text Figure 4b), where the pl-WG is extended to x = 10 μm, and the ph-WG is extended to x = 20 μm, we present the measurement results for Sample 2 in Figure S2a. Sample 2 also features the ph-WG extended to x = 20 μm. However, the Au strip is terminated at the right boundary of the TJ area. It should be noted that the TJ area of Sample 2 exhibits more non-uniformity in the light emission, primarily due to hBN barrier leakage. Nevertheless, the $I(V_b)$ curve (in Figure S2b) still exhibits the characteristic superlinear behavior associated with tunneling. Hence, we believe that despite the sample imperfections, the light emission of this device is still attributed to the IET process. To mitigate the impact of the sample imperfections, we normalize the integrated intensities by dividing them by the average current (see main text Figure 4c).

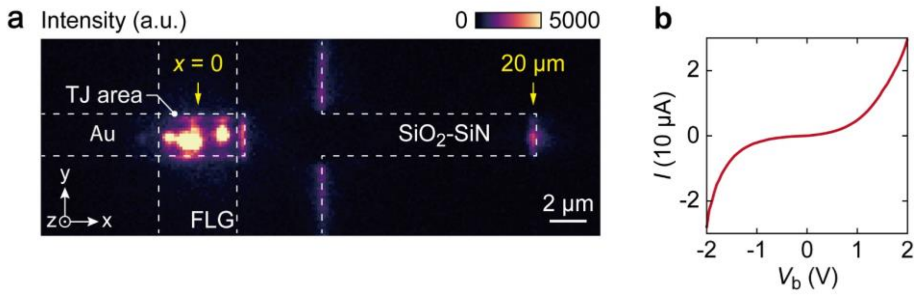

Figure S2. Data for Sample 2, where the ph-WG is extended to x = 20 μm without the Au strip extension. a) Light emission image. b) $I(V_\mathrm{b})$ curve.

Section S4. Coupling efficiencies for M1 and M2

A. Theoretical coupling efficiency

From the $\Delta\Gamma$ spectra shown in main text Figure 1c, the normalized decay rate (Γ_i) into modes M1 (Γ_{M1}) and M2 (Γ_{M2}) for a given dipole energy ($\hbar\omega_0$, or wavelength λ_0) can be obtained by integrating the range in which the value decays to 5% of the peak value. Whereas for photon radiation (Γ_{rad}), it is integrated from 0 to the index of glass ($k_{||}/k_0$ = 1.45). The spectral efficiencies (Υ_i) for the three different contributions can be given by[2]

$$\Upsilon_i = \left(\frac{1}{\hbar\omega_0}\right)\frac{\pi}{12\varepsilon_0}\rho_0\frac{P_\mathrm{I}(\omega)}{\gamma_{el}}\Gamma_i \qquad \text{(S2)}$$

where $\rho_0 = \omega_0^2/(\pi^2 c^3)$ is the LDOS for free space, γ_{el} is the rate of elastic electron tunneling, $P_\mathrm{I}(\omega)$ is the power spectral density of IET.[3] Note that $P_\mathrm{I}(\omega)$ is calculated from the $I(V_\mathrm{b})$ curve in main text Figure 3c, which guarantees the quantum cutoff limit and maintains the upper energy limit of IET dipole at eV_b. The relative coupling efficiencies can be determined by calculating the ratio of a specific mode (Υ_i) to the total optical outcoupling (Υ_{opt}) given by

$$\eta_{\mathrm{M1}}^{\mathrm{theo}} = \frac{\Upsilon_{\mathrm{M1}}}{\Upsilon_{\mathrm{opt}}} = \frac{\Upsilon_{\mathrm{M1}}}{\Upsilon_{\mathrm{rad}} + \Upsilon_{\mathrm{M1}} + \Upsilon_{\mathrm{M2}}} \qquad \text{(S3)}$$

$$\eta_{\mathrm{M2}}^{\mathrm{theo}} = \frac{\gamma_{\mathrm{M2}}}{\gamma_{\mathrm{opt}}} = \frac{\gamma_{\mathrm{M2}}}{\gamma_{\mathrm{rad}} + \gamma_{\mathrm{M1}} + \gamma_{\mathrm{M2}}}$$

where γ_{M1}, γ_{M2}, and γ_{rad} are the spectral efficiencies of M1, M2, and photon radiation, respectively. By integrating the relative coupling efficiencies over the energy range while considering the power spectral density distribution, the estimated values for M1 and M2 are (62.77 ± 1.74) % and (29.07 ± 0.72) %, respectively.

B. Experimental coupling efficiency

From the spectra (in main text Figure 5a) collected at *x* = 0, 10, and 20 μm, the emitted power from these three points (P_0, P_{10}, and P_{20}) are obtained as a function of V_{b}. Propagation losses within the pl-WG (with a length of L_{Au} = 10 μm and the width of W_{Au} = 2 μm) are corrected for P_{10} and P_{20}, by multiplying a factor of exp $(L_{\mathrm{Au}}/l_{\mathrm{p}})$, where l_{p} is the propagation length for a guided mode (refer to main text Figure 2b), considering the additional loss caused by the 1 nm-thick Ti layer. The power coupled to all in-plane directions is accounted for by including a factor of emission angle, $\tan^{-1}$ $(W_{\mathrm{Au}}/2L_{\mathrm{Au}})/180°$. The relative coupling efficiency for M1 and M2 can be given by

$$\eta_{\mathrm{M1}}^{\mathrm{expt}} = \frac{P_{10} \times \exp\left(\frac{L_{\mathrm{Au}}}{l_{\mathrm{p,M1}}}\right) \times \frac{\tan^{-1}\left(\frac{W_{\mathrm{Au}}}{2L_{\mathrm{Au}}}\right)}{180°}}{P_0 + P_{10} \times \exp\left(\frac{L_{\mathrm{Au}}}{l_{\mathrm{p,M1}}}\right) \times \frac{\tan^{-1}\left(\frac{W_{\mathrm{Au}}}{2L_{\mathrm{Au}}}\right)}{180°} + P_{20} \times \exp\left(\frac{L_{\mathrm{Au}}}{l_{\mathrm{p,M2}}}\right) \times \frac{\tan^{-1}\left(\frac{W_{\mathrm{Au}}}{2L_{\mathrm{Au}}}\right)}{180°}}$$

$$\eta_{\mathrm{M2}}^{\mathrm{expt}} = \frac{P_{20} \times \exp\left(\frac{L_{\mathrm{Au}}}{l_{\mathrm{p,M2}}}\right) \times \frac{\tan^{-1}\left(\frac{W_{\mathrm{Au}}}{2L_{\mathrm{Au}}}\right)}{180°}}{P_0 + P_{10} \times \exp\left(\frac{L_{\mathrm{Au}}}{l_{\mathrm{p,M1}}}\right) \times \frac{\tan^{-1}\left(\frac{W_{\mathrm{Au}}}{2L_{\mathrm{Au}}}\right)}{180°} + P_{20} \times \exp\left(\frac{L_{\mathrm{Au}}}{l_{\mathrm{p,M2}}}\right) \times \frac{\tan^{-1}\left(\frac{W_{\mathrm{Au}}}{2L_{\mathrm{Au}}}\right)}{180°}} \tag{S4}$$

Section S5. Quantum efficiency plot of the EMCCD

Figure S3 depicts the quantum efficiency of the EMCCD (Andor iXon Ultra 897)[4] used to acquire our light emission images. The EMCCD exhibits a detection wavelength range from 300 to 1100 nm.

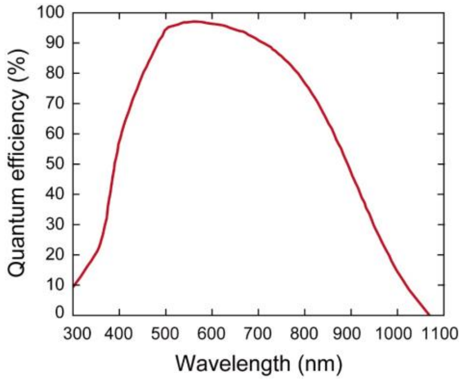

Figure S3. Detection efficiency plot of the EMCCD.

References

1. Novotny, L.; Hecht, B. *Principles of Nano-Optics*; Cambridge University Press, 2012.

2. Wang, Z.; Kalathingal, V.; Hoang, T. X.; Chu, H.-S.; Nijhuis, C. A., Optical Anisotropy in van der Waals materials: Impact on Direct Excitation of Plasmons and Photons by Quantum Tunneling. *Light: Science & Applications* **2021,** *10* (1), 230.

3. Rogovin, D.; Scalapino, D. J., Fluctuation phenomena in tunnel junctions. *Annals of Physics* **1974,** *86* (1), 1-90.

4. Andor support resources. https://andor.oxinst.com/products/emccd-cameras (accessed 2023-12-14).
